# Juvenile Greylag Geese (*Anser anser*) Discriminate between Individual Siblings

**DOI:** 10.1371/journal.pone.0022853

**Published:** 2011-08-03

**Authors:** Isabella B. R. Scheiber, Aileen Hohnstein, Kurt Kotrschal, Brigitte M. Weiß

**Affiliations:** 1 Konrad Lorenz Forschungsstelle für Ethologie, Grünau, Austria; 2 Department of Behavioural Biology, The University of Vienna, Vienna, Austria; 3 Institute of Biology, Humboldt University of Berlin, Berlin, Germany; 4 Institute of Biology, The University of Neuchâtel, Neuchâtel, Switzerland; University of Lethbridge, Canada

## Abstract

Social species that maintain individualised relationships with certain others despite continuous changes in age, reproductive status and dominance rank between group members ought to be capable of individual recognition. Tests of “true” individual recognition, where an individual recognises unique features of another, are rare, however. Often kinship and/or familiarity suffice to explain dyadic interactions. The complex relationships within a greylag goose flock suggest that they should be able to recognise individuals irrespective of familiarity or kinship. We tested whether six-week-old hand-raised greylags can discriminate between two of their siblings. We developed a new experimental protocol, in which geese were trained to associate social siblings with geometrical symbols. Subsequently, focals were presented with two geometrical symbols in the presence of a sibling associated with one of the symbols. Significant choice of the geometrical symbol associated with the target present indicated that focals were able to distinguish between individual targets. Greylag goslings successfully learned this association-discrimination task, regardless of genetic relatedness or sex of the sibling targets. Social relationships within a goose flock thus may indeed be based on recognition of unique features of individual conspecifics.

## Introduction

Groups of social animals are structured by cohesion and mutual interactions within pairs, families, and/or matrilines or similar alliances [1]. This social structure suggests a capacity for both kin recognition and individual recognition [2], which, in turn, has implications for the evolution of social behaviour [2]–[5]. Kin recognition is an animal's ability to distinguish between close kin and non-kin (e.g. [6] and references therein), and there is ample evidence for preferential allocation of aid to kin in birds (reviewed in [7]). In general, there are three major domains of kin recognition: parent-offspring recognition [8], offspring-parent recognition and sibling recognition [9], with most attention in birds devoted to parent-offspring recognition in colonially breeding birds (e.g. [10]). Avian sibling recognition has received much less consideration [9]. Helping kin, however, does not necessitate individual recognition, because helpers may support individuals with whom they are most familiar as shown in tree swallows (*Tachycineta bicolor*) and barn swallows (*Hirundo rustica*, [11]), or which can be found in specific locations as shown in the Caspian tern (*Sterna caspia*, [12]).

The social behaviour in many animal species appears to be finely tuned to the identity of the interactants; often members of animal societies behave differently towards one another not only depending on kinship, but also on sex, dominance rank, reproductive condition and their previous history of interaction [13]. When multiple individuals with differing intentions interact with one another repeatedly, recognition of unique individual features, *i.e.* “true” individual recognition, seems a valuable skill, and is thought to require specific cognitive adaptations [10]. Also, the occurrence of stable, long-term biparental care suggests that many, if not most, organisms can individually identify their mates [14].

Many models of social interactions have individual recognition as a crucial assumption, but most tests of individual recognition did not allow to distinguish between recognition of actual individual-related cues and class-level recognition, i.e. cues related to classes such as familiarity [15], location [12] or kinship [16]. Less restrictive definitions suggest that also class-level recognition, familiarity in particular, should be regarded as individual recognition (e.g. [17]), but “true” individual recognition is generally regarded as a form of recognition in which a receiver learns the unique, individual-distinctive features of the signaller and associates these characteristics with individual-specific information about it (e.g. [18], see also [19] for a review). While such true individual recognition is difficult to test, a few studies do provide evidence that animals like paper wasps, elephants or rats, indeed recognise the unique features of individual group members [18], [20], [21] in the absence of class-related cues.

Greylag geese are socially complex [22], [23] and display a variety of sophisticated social interactions [24]–[33] , which are thought to favour individual recognition. We, therefore, hypothesise that greylag geese are capable of true individual recognition and that this ability develops early in life. To avoid alternative explanations, such as familiarity or kinship, we tested hand-raised sibling groups to determine if focals truly do discriminate between individuals of equal familiarity and relatedness. To answer this question we modified a two-way choice experimental protocol based on an association-discrimination protocol (see Methods), which allows for testing individual recognition between individuals from the same social class.

## Methods

### Ethical Statement

The conducted study complies with all current Austrian laws and regulations concerning the work with wildlife (Oberoesterreichische Schonzeitverordnung 2007 - LGBI.Nr. 72/2007). For hand-raising, we collected eggs from four nests of an Italian greylag goose population of the Regional Natural Reserve of Valle Canal Novo, Udine (Collection permit: RAF 13/12.5/15835). Sex determination was performed under Animal Experiment License BMWF-66.006/0010-II/10b/2010. No other manipulations of the geese, which would have required additional licenses, were conducted.

### Animals

A free-flying, non-migratory flock of greylag geese was introduced into the valley of the river Alm, Austria, by Konrad Lorenz and co-workers in 1973 [25]. The flock is unrestrained but habituated to the presence of humans and is provisioned with pellets and grain twice daily. At the time of this study, the flock consisted of 150 birds individually marked with coloured leg bands, whose life histories have been monitored continuously. About 25% of the individuals were hand-raised by human foster parents under near-natural conditions. Detailed procedures of the long-standing hand-raising tradition of the KLF are described elsewhere [34]. Hand-raised goslings are in contact with the flock from hatching on and fully integrate into the flock after fledging. They establish pair bonds and raise offspring indistinguishable from the goose-raised geese, but maintain a life-long confidence towards familiar humans [34].

We experimentally tested 15 hand-raised greylag goslings (eight females, seven males) from three sibling groups raised in 2009. Detailed information about the focal individuals as well as the tested sibling dyads is given in Table 1. Eggs for groups A and B were collected from four nests of an Italian greylag goose population of the Regional Natural Reserve of Valle Canal Novo, Udine, therefore, not all individuals of one group are genetically related or of the exact same age (see Table 1). However, goslings raised together perceive one another as family [35]. Eggs for group C were collected from one local nest; goslings of group C were all genetically related and of the same age. Eggs were incubated and hatched in a commercial incubator (Fa. Hemel Brutgeräte®) at the Konrad Lorenz Research Station (KLF). Immediately after hatching, goslings were individually marked with grey leg bands on their left leg, labelled with a letter (A, B, or C) for the three groups as well as an individual number (01–05). These leg bands were used throughout the course of the experiment; however, bands were replaced several times due to the goslings' growth. The final individual colour band combination, which marks the goslings individually within the flock and would give a cue for individual recognition, was affixed shortly before fledging, at a time when this experiment was already terminated.

**Table 1:** Detailed information of the 15 focal individuals, which participated in the sibling recognition experiment as well as the randomly chosen sibling targets.

Family Group	Individual	Name Abbreviated	Hatch Date (2009)	Genetic sibling group	Sex	Sibling targets	Sex targets
A	1	KOR	April 10^th^	1	M	KAM, KRA	F-F
A	2	KAM	April 10^th^	1	F	**KOR, MIR**	M-M
A	3	ING	April 10^th^	1	F	MIR, KRA	M-F
A	4	MIR	April 11^th^	1	M	**KAM, ING**	F-F
A	5	KRA	April 18^th^	2	F	**KOR, ING**	M-F
B	6	PRO	April 10^th^	3	M	**PER, GAI**	M-F
B	7	PER	April 11^th^	3	M	PRO, MED	M-M
B	8	GAI	April 11^th^	3	F	PRO, KRO	M-F
B	9	MED	April 12^th^	4	M	PER, KRO	M-F
B	10	KRO	April 12^th^	4	F	GAI, MED	M-F
C	11	FRI	April 15^th^	5	F	**FRZ, EDE**	M-M
C	12	FRZ	April 15^th^	5	M	**FRI, HIL**	F-F
C	13	HIL	April 15^th^	5	F	**FRI, BOL**	F-F
C	14	BOL	April 15^th^	5	F	**HIL, EDE**	F-M
C	15	EDE	April 15^th^	5	M	**FRI, BOL**	F-F

Sibling targets marked in bold represent targets genetically related to one another.

All 15 individuals had participated in spontaneous choice tests when they were three and ten days old, to determine at which point in time they prefer siblings to non-siblings. They had no prior experience with the experimental set-up used for the sibling recognition tests.

### Training procedures

#### Preliminary Training

Subjects were trained to retrieve a favoured food item, *i.e.* a small piece of bread, from a grey cup (length 7.5 cm×width 7.5 cm×height 7.5 cm) by pushing or pulling off a square grey lid (8.5×8.5 cm). After this, they were offered two cups with grey lids. They were allowed to open both, but only one cup was baited. When birds reliably opened both cups, a second training step (learning phase) followed.

#### Experimental Set-Up

The learning phase and experiments were conducted in an outdoor arena (length 4.85 m×width 2.25 m×height 1.30 m) that allowed testing without interference from other geese and did not allow the other geese participating in the experiments to watch the trials. This arena was built adjacent to the porch of one of the hand-raiser's huts and connected with a door to the porch. The family group to be tested could be kept on the porch, and focals and their respective targets could be easily led in and out of the arena. Two targets per focal, matched in dominance, were chosen and one of eight geometrical symbols (*i.e.* triangle, diagonal line, double line, circle, star, plus, three dots, the letter ‘S’) was assigned randomly to either target. Sex of the 15 individuals was unknown when the experiment was performed but was later determined using molecular markers following the protocol of Griffiths [36]. Although both targets were equally familiar to the respective focal, their random selection resulted in seven mixed-sex and eight same-sex (male-male N = 3; female-female N = 5) groups as well as some genetically related target groups (N = 9 out of 15). Genetic relatedness of nest mates was later confirmed with microsatellite markers [37]. Therefore, some target groups may have provided kin cues, whereas others could have potentially been distinguished by sex differences.

#### Learning Phase

Training with geometrical symbols started May 27^th^, 2009. In a first step, the focals were given the chance to learn an association between target and symbol. One of the targets was placed together with the focal in the arena. The focal was given one cup, baited with one bread cube, and covered with the lid that carried the geometrical symbol associated with the target present. The focal was allowed to open the cup 25 times in the presence of either of two target (5 presentations / day / target for a total of five days). Presentations were spread over the course of one day with at least 10 minutes between presentations. After this training the focal advanced to the recognition tests.

#### Individual Recognition Phase

Individual recognition training was performed between June 18^th^ and July 28^th^, 2009. Tests were conducted daily with half a session (eight trials) taking place in the early morning (8:00 AM to 9:30 AM), and the second half of the session (eight trials) taking place in the later morning (11:00 AM to 12:30 PM). Experiments were conducted over 41 days, at which point the experiment had to be terminated due to time constraints of the experimenter (AH), who conducted all formal training and tests. During the individual recognition training focals were presented with two cups, covered with the geometrical symbols assigned to the focal's two target siblings. Only one of the targets was present and only the cup with the symbol associated with the present target was baited. To reliably open the baited cup focal individuals had to learn to associate the presence of a particular target with the corresponding geometrical symbol.

As all individuals per family group were focals and targets, the order in which they entered the arena as focals was randomised for each session. Similarly the order of which target per focal entered the arena was randomised. Before each trial the experimenter baited one of the cups outside the visual field of the focal. After geese greeted one another, which ensured that the focal had seen the target, the focal was presented with the two cups. The position of the baited cup was randomised throughout the session. Both cups were shown to the focal for one second by the experimenter and were then placed in front of the focal approximately 40 cm apart. Immediately after placing the cups in front of the focal, the experimenter removed both hands simultaneously. The focal goose was then allowed to open one cup only. To avoid interference with the focal's choice, the target was blocked from access to the cups by the experimenter placing herself between focal and target. The order of the targets was randomised under the restriction that the same target was not in the arena in more than three successive trials. When a focal performed above chance, that is, reached a criterion of 13 or more correct responses in each of two consecutive sessions, we assumed that it was capable of discriminating the two targets and the experiment was terminated.

### Statistical Analyses

To determine, whether individual goslings passed the sibling recognition task, we applied binomial tests. On a group level we compared the number of correct versus incorrect choices in the last two sessions an individual participated in with paired t-tests. In order to determine performance in the recognition task, we conducted a generalized linear mixed model (GLMM) applying the restricted maximum likelihood procedure (REML). We constructed the GLMM with choice (correct or wrong) in the 32 trials of the last two sessions as the binominal response variable and ‘sex of focal’, ‘same sex or mixed sex target groups’, ‘genetic or non-genetic target groups’ and ‘family group’ as fixed terms. In the model we included the individual as a random factor to account for repeated measures within individuals. We sequentially deleted fixed terms in order of decreasing significance; only terms with p<0.05 remained in the final model [38], [39]. Excluded terms were re-entered one by one into the final model to confirm that they did not explain a significant part of the variation. Data were analysed using Sigma Stat 3.5 (Systat Software, San Jose, CA, U.S.A.) and GenStat Release 10.1 (Lawes Agricultural Trust, 2007). Results are two tailed with p set to 0.05. Means and standard errors are given throughout.

## Results

**Figure 1:**
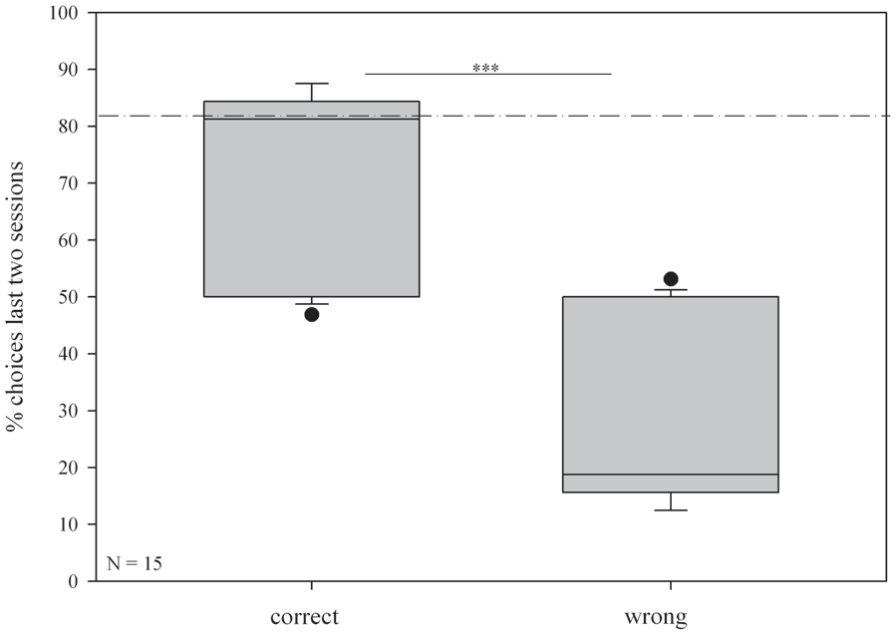
Percentage of correct and wrong choices of all 15 focal individuals on the penultimate and ultimate day of the recognition task. 81.25% and above marks a performance above chance (dotted line). Asterisks mark significant differences: *p<0.001*.

Overall, geese were capable of individually recognising their sibling targets (mean choices ± SE: correct: 23.53±1.31; wrong: 8.47±1.31; paired t-test: N = 15, t_28_ = 8.16, p<0.001, Figure 1). On an individual level, 10 of 15 goslings passed the criterion in the recognition task as described above within the allotted 41 days (Table 2). The ten individuals passed in a mean time of 33.5 days (± SE: 2.33; range: 20–41 days). Although only 20% of the males, but 70% of the females passed the recognition task, there was no significant statistical difference detectable (GLMM, Table 3).

**Table 2:** Performance of the 15 focal individuals on the penultimate and ultimate days when goslings passed the task, or of the 40^th^ and 41^st^ days of the individuals that did not pass (DNP) the task in the allotted time.

Family Group	Individual	Name Abbreviated	Sex	Correct Choices Penultimate day	Correct Choices Ultimate day	Days to Complete
A	1	KOR	M	9	6	DNP
A	2	KAM	F	**13**	**13**	41
A	3	ING	F	**13**	**14**	27
A	4	MIR	M	**13**	**15**	37
A	5	KRA	F	**14**	**13**	37
B	6	PRO	M	9	7	DNP
B	7	PER	M	12	10	DNP
B	8	GAI	F	7	9	DNP
B	9	MED	M	7	9	DNP
B	10	KRO	F	**13**	**13**	40
C	11	FRI	F	**13**	**14**	24
C	12	FRZ	M	**13**	**13**	40
C	13	HIL	F	**13**	**13**	36
C	14	BOL	F	**14**	**14**	33
C	15	EDE	M	**14**	**13**	20

Session marked in bold indicate performance above chance (Binomial tests P<0.05).

**Table 3:** Statistical results of the generalized linear mixed model (GLMM) to determine possible influences on the performance in the recognition task.

Fixed term	Full fixed model			Final Model		
	Wald statistics	ndf	p	Wald statistics	ndf	p
Sex focal	1.57	1	0.241	*1.94*	*1*	*0.190*
Same vs. mixed sex target groups	0.02	1	0.878	*0.32*	*1*	*0.582*
Genetic vs. non-genetic target groups	0.67	1	0.434	*0.51*	*1*	*0.489*
Family group (A–C)	1.47	2	0.504	**9.08**	**2**	**0.032**

The binomial response variable was correct/wrong choice in the ultimate 32 trials of the 15 focal individuals. For the full model, results of all tested fixed terms are given. For the final model, results of terms that remained in the final model are given in bold, and results of excluded terms when individually re-entered into the final model are given in italics.

Importantly, goslings were capable of distinguishing individual siblings regardless of whether target groups consisted of two siblings of the same sex (paired t-test: N = 7, t_12_ = 4.87, p = 0.003) or different sex (paired t-test: N = 8, t_14_ = 3.39, p = 0.012). Performance did not differ between same sex and mixed sex target groups (GLMM, Table 3). Similarly, they discriminated between their siblings regardless of whether these were genetically related (N = 9, t_16_ = 11.06, p<0.001) or not genetically related (paired t-test: N = 6, t_10_ = 2.78, p = 0.019). Focals did not perform significantly better or worse when the target group consisted of two genetic siblings (GLMM, Table 3).

**Figure 2:**
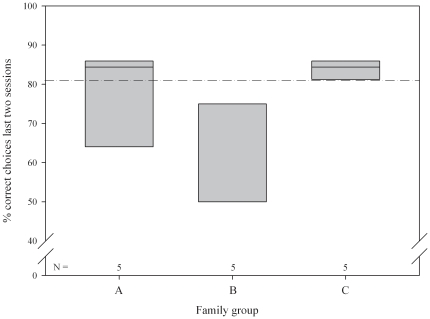
Percentage of correct choices of family groups A, B, and C on the penultimate and ultimate day of the recognition task.

The three siblings groups differed in their performance (Figure 2): in sibling group A 80% passed (4/5 individuals, mean correct choices ± SE: 24.6±2.42), in sibling group B 20% passed (1/5 individuals, mean correct choices ± SE: 19.2±2.06) and in sibling group C 100% passed (5/5 individuals, mean correct choices ± SE: 26.8±0.37, GLMM Table 3).

## Discussion

Our modified experimental design, where geese had to associate geometrical symbols with target individuals, proved to be a suitable tool for testing individual recognition in geese and allowed us to show that greylag goslings can distinguish between individual siblings when approximately 12 weeks old. Thereby, the individuals to be discriminated were equally familiar to the focals, ruling out familiarity as a means of differentiating between individuals. Similarly, focals passed the task even if siblings were of the same sex and genetically related, thereby providing sound evidence that discrimination in this task was not based on other indirect cues like kinship or sex differences, but indeed on recognition of their siblings' unique individual features. Furthermore, performance in the task did not improve if sex or kin-related cues were also available, suggesting that discrimination is primarily based on individually unique features even if more general cues are available. Which individual features were used was not examined in this experiment, but acoustic and/or visual cues are likely candidates, as these two seem to be the most astute sensory channels in birds [40]. Follow-up experiments can tackle this question by presenting either only selected visual or auditory cues.

Our test set up differs from other recognition test procedures like the habituation-dishabituation paradigm by not only asking if two individuals are perceived as different, but by asking focal animals to respond differently (i.e. choose a different symbol) to individuals that are equally familiar, equally related and of the same sex. This set-up not only necessitates individual recognition, but also the association between an individual that is present, yet not actively involved in the task, and a specific geometric symbol. This may be one reason why some individuals failed to reach the test criterion: focals may have been unable or too slow to form the association between a sibling's presence and a geometric symbol in the given time frame. Alternatively, it is possible that these individuals were indeed unable to distinguish between their siblings or that they were incapable of recognising the symbols. Other experiments showed that geese are per se capable of discriminating geometrical symbols like those used in this study, but that learning to discriminate symbol pairs may take up to three times longer than discriminating e.g. colour pairs (BMW, unpubl.).

Sex differences in problem solving tasks and different cognitive strengths are known from humans and non-human mammals [41], [42]. For instance, men generally excel in spatial skills, while women usually perform better in tests which require rapid matching or identification of designated stimuli [41] as well as tasks in the social context [43]–[46]. In greylag geese, the long-term bonds among female relatives and the benefits of social support to females make individual recognition particularly beneficial for females [22], [24], [26], [28], [29], [32], [47]. At a first glance, female geese indeed seemed to perform better than males, as four of the five individuals who failed the task were male. The results of the GLMM, however, did not support this impression and it remains to be determined if this was an effect of the relatively low number of individuals tested, or if sex differences in individual recognition abilities of geese are indeed absent.

Finally, we found a difference in performance between the three sibling groups. One reason may be that sibling group C was raised by the experimenter (AH). If this influenced performance in the task, we would expect similar performances of groups A and B, as these were both raised by other foster parents and as such equally familiar to the experimenter (AH). However, individuals of group A performed similarly well as those of group C, while the goslings of group B were considerably worse. To some extent this may be due to a higher number of males in group B, although sex differences probably cannot fully explain the difference between the groups. Additionally or alternatively, the groups may have differed in the genetic prerequisites underlying cognitive abilities (‘genes of cognitive abilities’, reviewed in [48], [49]): three of the four goslings, which failed the task in family group B were genetic siblings, similarly to the five of five that passed in group C. Lastly, another possibility for the poor performance of the B group may be non-genetic parental effects. Hormonal influences in the eggs or differences in “parenting style” of the human foster parent may have influenced physiology and/or behaviour [50], [51], e.g. the degree of competition within brood mates ([30] , see also reviews in [52], [53]. At present, however, we cannot support or reject any of these possibilities.

In conclusion, our findings demonstrated that free-ranging greylag geese are capable of true individual recognition. With a new experimental design, which is based on an association – discrimination protocol, we were able to ask our study organisms about whether they can actually identify individuals from the same social class and with whom they are equally familiar. Individual recognition presumably is a widespread skill throughout the vertebrates, which – with the appropriate methods – might also be demonstrated in various other social vertebrate species.

## References

[1] Hinde R (1983). Primate Social Relationships - An Integrated Approach.

[2] Rendall D, Rodman PS, Emond RE (1996). Vocal recognition of individuals and kin in free-ranging rhesus monkeys.. Anim Behav.

[3] Hamilton WD (1963). The evolution of altruistic behavior.. Am Nat.

[4] Hamilton WD (1964a). The genetical evolution of social behavior I.. J Theor Biol.

[5] Hamilton WD (1964b). The genetical evolution of social behavior II.. J Theor Biol.

[6] Sayigh LS, Tyack PL, Wells RS, Solow AR, Scott MD (1998). Individual recognition in wild bottlenose dolphins: a field test using playback experiments.. Anim Behav.

[7] Komdeur J, Hatchwell BJ (1999). Kin recognition: function and mechanism in avian societies.. TREE.

[8] Draganoiu TI, Nagle L, Musseau R, Kreutzer M (2006). In a songbird, the black redstart, parents use acoustic cues to discriminate between their different fledglings.. Anim Behav.

[9] Nakagawa S, Waas JR (2004). ‘O sibling, where art thou?’ - a review of avian sibling recognition with respect to the mammalian literature.. Biol Rev.

[10] Beecher MD (1988). Kin recognition in birds.. Behav Genet.

[11] Burtt EHJ (1977). Some factors in the timing of parent-chick recognition in swallows.. Anim Behav.

[12] Shugart GW (1977). The development of chick recognition by adult Caspian terms.. P Colon Waterbird.

[13] Seyfarth RM, Cheney DL, de Waal FBM, Tyack PL (2003). The structure of social knowledge in monkeys.. Animal Social Complexity.

[14] Tibbetts EA, Dale J (2007). Individual recognition: it is good to be different.. TREE.

[15] Godard R (1991). Long-term memory of individual neighbours in a migratory songbird.. Nature.

[16] Tang-Martinez Z, Bixler A (2009). Individual discrimination by odors in sibling prairie voles.. J Chem Ecol.

[17] Steiger S, Müller JK (2008). ‘True’ and ‘untrue’ individual recognition: suggestion of a less restrictive definition.. TREE.

[18] Tibbetts EA (2002). Visual signals of individual identity in the wasp *Polistes fuscatus*. P R Soc Lond, Ser.. B: Biol Sci.

[19] Tricarico E, Borrelli L, Gherardi F, Fiorito G (2011). I know my neighbour: individual recognition in *Octopus vulgaris*.. PLoS One.

[20] Bates LA, Sayialel KN, Njiraini NW, Poole JH, Moss C (2008). African elephants have expectations about the locations of out-of-sight family members.. Biol Lett.

[21] Gheusi G, Goodall G, Dantzer R (1997). Individually distinctive odours represent individual conspecifics in rats.. Anim Behav.

[22] Weiß BM, Kotrschal K, Frigerio D, Hemetsberger J, Scheiber IBR, Ramirez RN (2008). Birds of a feather stay together: extended family bonds and social support in greylag geese (*Anser anser*).. Family Relations: Issues and Challenges.

[23] Kotrschal K, Scheiber IBR, Hirschenhauser K, Kappeler PM (2010). Individual performance in complex social systems: the greylag goose example.. Animal Behaviour: Evolution and Mechanisms.

[24] Frigerio D, Weiß BM, Kotrschal K (2001). Spatial proximity among adult siblings in Greylag Geese (*Anser anser*): evidence for female bonding?. Acta Ethol.

[25] Lorenz K (1988). Hier bin ich - wo bist Du? Ethologie der Graugans.

[26] Scheiber IBR, Kotrschal K, Weiß BM (2009). Benefits of family reunions: social support in secondary greylag goose families.. Horm Behav.

[27] Weiß BM, Kotrschal K (2004). Effects of passive social support in juvenile Greylag Geese (*Anser anser*): a study from fledging to adulthood.. Ethology.

[28] Scheiber IBR, Weiß BM, Frigerio D, Kotrschal K (2005). Active and passive social support in families of Greylag Geese (*Anser anser*).. Behaviour.

[29] Scheiber IBR, Kotrschal K, Weiß BM (2009b). Serial agonistic attacks by greylag goose families (*Anser anser*) against the same opponent.. Anim Behav.

[30] Kalas S (1977). Ontogenie und Funktion der Rangordnung innerhalb einer Geschwisterschar von Graugänsen (*Anser anser* L.).. Z Tierpsychol.

[31] Hohnstein A (2010). Individualerkennung bei Graugänsen.

[32] Kotrschal K, Hemetsberger J, Weiß BM, Vasey P, Sommer V (2006). Making the best of a bad situation: homosociality in male greylag geese.. Homosexual Behaviour in Animals: An Evolutionary Perspective.

[33] Weiß BM (2000). Social Support in Juvenile Greylag Geese (*Anser anser*).

[34] Hemetsberger J, Scheiber IBR, Weiß BM, Frigerio D, Kotrschal K (2010). Influence of socially involved hand-raising on life history and stress responses in greylag geese.. Interact Stud.

[35] Kalmbach E (2006). Why do goose parents adopt unrelated goslings? A review of hypotheses and empirical evidence, and new research questions.. Ibis.

[36] Griffiths R, Double MC, Orr K, Dawson RJG (1998). A DNA test to sex most birds.. Mol Ecol.

[37] Weiß BM, Poggemann K, Olek K, Foerster K, Hirschenhauser K (2008). Isolation and characterization of microsatellite marker loci in the greylag goose (*Anser anser*).. Mol Ecol Resources.

[38] Galwey NW (2006). Introduction to Mixed Modelling: Beyond Regression and ANOVA.

[39] Garamszegi LZ, Calhim S, Dochtermann NA, Hegyi G, Hurd PL (2009). Changing philosophies and tools for statistical inferences in behavioral ecology.. Behav Ecol.

[40] Sturkie PD (1986). Avian Physiology, 4th edn.

[41] Kimura D (2002). Sex hormones influence human cognitive pattern.. Neuroendocrinol Lett.

[42] Gaulin SJC, FitzGerald RW, Wartell MS (1990). Evolution and development of sex differences in spatial ability and activity in two vole species.. J Comp Psychol.

[43] Baron-Cohen S (2004). The Essential Difference.

[44] Halpern DF (2004). A cognitive-process taxonomy for sex differences in cognitive abilities.. Curr Direct Psychol Sci.

[45] Halpern DF (2000). Sex Differences in Cognition Abilities, 3rd edn.

[46] Kimura D (2000). Sex and Cognition.

[47] Swoboda R (2006). Social modulation of immuno-reactive corticosterone metabolites in goslings from hatching to fledging: ontogeny of social support in greylag geese (*Anser anser*).

[48] Morley KI, Montgomery GW (2001). The genetics of cognitive processes: candidate genes in humans and animals.. Behav Genet.

[49] Wassermann EA, Zentall TR (2006). Comparative Cognition in Animals: Experimental Explorations of Animal Intelligence.

[50] Daisley NJ, Bromundt V, Möstl E, Kotrschal K (2005). Enhanced yolk testosterone influences behavioral phenotype independent of sex in Japanese quail chicks *Coturnix japonica*.. Horm Behav.

[51] Maestripieri D (1999). The biology of human parenting: insights from non-human primates.. Neurosci Biobehav Rev.

[52] Drummond H (2006). Dominance in vertebrate broods and litters.. Quart Rev Biol.

[53] Hudson R, Trillmich F (2008). Sibling competition and cooperation in mammals: challenges, developments and prospects.. Behav Ecol Sociobiol.

